# Supplementary material for: Differential Gene Expression in Brain and Liver Tissue of Wistar Rats after Rapid Eye Movement Sleep Deprivation
Source: Clocks Sleep. 2020 Oct 23;2(4):442–65. doi: 10.3390/clockssleep2040033 (PMC7711450; doi:10.3390/clockssleep2040033)
Supplement: Supplementary file 1 [file clockssleep-02-00033-s001.zip › Supplimentary Tables.pdf]

Supplementary materials:

Table S1: List of real time PCR gene primers/probes used in microarray validation experiments

S. N	Primer/probe	Primer/probe sequence ID's
1	Alpha 1 inhibitor 3	Rn00440636-m1
2	Interleukin-1b	Rn00580432_m1
3	TP53	Rn00755717_m1
4	Bcl2	Rn99999125_m1
5	Bax	Rn02532082_g1
6	Bid	Rn01459517_m1
7	G6pD	Rn01529640_g1
8	GAPDH	Rn01749022_g1
9	Master mix	Cat. 4304437

Table S2: List of genes upregulated in brain (A), downregulated in brain (B), upregulated in liver (C), downregulated in liver (D).

Table S2A: List of genes upregulated in brain

Probe Set ID	Control Brain	Sleep Deprived Brain	Log Fold Change	Fold Change	Entrez ID	Gene Symbol	Accession
10701797	5.09	5.98	0.89	1.85	680891	Sf3b5	NM_001126092
10701825	4.66	5.69	1.03	2.05	361453	Deadc1	NM_001115028
10702286	6.52	7.54	1.01	2.02	65139	Rps12	NM_031709
10702306	2.93	3.79	0.86	1.81	252856	Tcf21	NM_001032397
10702361	3.80	4.81	1.01	2.01	155430	Hey2	NM_130417
10702592	7.46	8.37	0.90	1.87	683872	LOC683872	ENSRNOT00000031807
10704411	6.75	7.69	0.94	1.92	29287	Rps19	NM_001037346
10704452	4.48	5.53	1.05	2.08	361514	Meis3	NM_001108472
10705414	6.97	8.33	1.35	2.55	361528	Ttc9b	NM_001108478

10705511	9.06	10.12	1.06	2.08	140655	Rps16	ENSRNOT0 0000026576
10705753	5.28	6.17	0.89	1.85	114004	Ppp1r14a	NM_130403
10706571	5.53	6.71	1.18	2.27	292876	Josd2	NM_001106 256
10710771	7.00	7.97	0.97	1.96	117053	Rps15a	NM_053982
10713538	5.99	6.89	0.90	1.87	24913	Pla2g16	NM_017060
10716085	6.70	7.84	1.13	2.19	65139	Rps12	NM_031709
10716373	3.40	4.45	1.05	2.07	25702	Pnlip	NM_013161
10717434	7.73	8.83	1.10	2.15	287417	Rpl26	NM_001105 788
10722005	4.04	5.20	1.16	2.23	83535	Kcnj11	NM_031358
10722084	7.07	8.13	1.05	2.08	365243	Sergef	ENSRNOT0 0000015352
10723233	7.22	8.45	1.23	2.34	29286	Rps17	NM_017152
10725078	3.48	4.50	1.02	2.02	499249	Plekha7	NM_001144 861
10726477	3.85	4.94	1.09	2.13	309095	Nkx6-2	NM_001107 558
10728312	6.40	7.34	0.94	1.92	293702	Fkbp2	NM_001134 429
10731729	8.40	9.28	0.88	1.84	363248	Rpl37a	NM_001100 994
10732439	4.86	6.28	1.41	2.66	685451	Gng13	NM_001135 918
10733888	4.80	5.92	1.12	2.17	287336	Olr1448	NM_001000 019
10734817	7.35	8.39	1.05	2.07	287417	Rpl26	NM_001105 788
10736053	9.03	9.98	0.94	1.92	81770	Rpl37	NM_031106
10736232	8.78	9.71	0.93	1.90	360572	Rpl23a	NM_001108 283
10737445	9.98	10.97	0.99	1.98	688136	LOC688136	XM_001081 257
10738657	5.82	6.80	0.98	1.98	303577	Acbd4	NM_001012 013
10739395	9.56	10.47	0.91	1.88	689284	Rpl38	NM_001077 592
10739994	2.84	3.86	1.02	2.03	303735	Rnf213	ENSRNOT0 0000004904
10741529	4.91	5.93	1.02	2.03	287151	Metrn	NM_001009 962
10741756	7.65	9.22	1.57	2.98	25632	Hba-a2	NM_013096
10741778	7.39	8.85	1.45	2.74	25632	Hba-a2	NM_013096
10745595	2.70	3.65	0.95	1.94	303378	Slfn8	NM_001013 970
10746952	7.41	8.31	0.90	1.87	57809	Rpl35a	NM_021264
10749058	6.94	7.91	0.96	1.95	294282	Rps18	NM_213557
10749061	4.93	5.87	0.93	1.91	287835	Galk1	NM_001008 282
10751767	4.53	5.54	1.02	2.03	27139	Rps26	NM_013224
10752146	6.99	7.86	0.87	1.82	117053	Rps15a	NM_053982
10753005	7.85	8.88	1.03	2.04	24786	Sod1	NM_017050

10756054	5.11	6.41	1.30	2.46	65131	Cldn5	NM_031701
10756455	4.06	4.97	0.91	1.88	140635	Flt3	NM_001100 822
10756546	9.69	10.57	0.88	1.84	690441	Atp5j2	ENSRNOT0 0000033537
10758457	4.37	5.35	0.98	1.97	29531	Hpd	NM_017233
10758777	2.96	4.04	1.08	2.12	192281	Oas1a	NM_138913
10760018	7.59	8.47	0.87	1.83	57809	Rpl35a	NM_021264
10761299	6.29	7.22	0.93	1.90	498173	Auts2	ENSRNOT0 0000044800
10762537	6.27	7.29	1.02	2.03	83620	Cit	NM_001029 911
10766797	3.83	4.74	0.90	1.87	364081	Irf6	NM_001108 859
10767371	8.85	9.86	1.01	2.01	27139	Rps26	NM_013224
10770117	4.99	5.99	1.01	2.01	289264	Grem2	NM_001105 974
10771040	5.24	6.17	0.93	1.90	689919	LOC689919	ENSRNOT0 0000061643
10771592	3.88	4.77	0.89	1.85	289501	Ankrd56	ENSRNOT0 0000002995
10772655	7.26	8.49	1.23	2.35	124323	Rps23	NM_078617
10772901	9.84	10.70	0.86	1.82	29507	Cox7a2	NM_022503
10772982	5.59	6.48	0.88	1.84	58927	Rpl36	NM_022504
10772984	5.59	6.48	0.88	1.84	58927	Rpl36	NM_022504
10774359	9.55	10.49	0.94	1.92	689284	Rpl38	NM_001077 592
10774372	7.68	8.72	1.04	2.06	691531	Rps28	NM_001105 730
10774894	8.32	9.57	1.25	2.37	124440	Rpl41	NM_139083
10776237	9.05	10.15	1.10	2.15	360572	Rpl23a	NM_001108 283
10778058	4.63	5.48	0.85	1.81	681091	Ccdc157	ENSRNOT0 0000006911
10778918	6.18	7.05	0.87	1.82	29287	Rps19	NM_001037 346
10780979	6.51	7.55	1.04	2.06	360572	Rpl23a	NM_001108 283
10781984	5.99	6.95	0.97	1.96	306157	Gpc5	NM_001107 285
10782041	5.80	7.10	1.31	2.48	306170	RGD1560050	ENSRNOT0 0000057567
10783353	6.26	7.12	0.86	1.82	29287	Rps19	NM_001037 346
10783357	2.60	3.51	0.92	1.89	290050	Olr1641	NM_001000 100
10784117	4.22	5.46	1.24	2.36	394266	Gjb2	NM_001004 099
10784512	2.85	3.79	0.94	1.92	498534	RGD1565212	NM_001109 103
10787085	2.59	3.71	1.12	2.17	25331	Mat1a	NM_012860
10788293	2.23	3.07	0.85	1.80	290757	F11	NM_001047 848

10790471	4.76	5.81	1.05	2.07	64347	Sncg	NM_031688
10790525	9.55	10.49	0.94	1.92	689284	Rpl38	NM_001077592
10791497	2.81	3.84	1.03	2.04	29395	Hmgb2	NM_017187
10792163	5.13	5.98	0.85	1.80	116636	Eif4ebp1	NM_053857
10795546	6.25	7.15	0.90	1.86	29287	Rps19	NM_001037346
10796677	9.55	10.41	0.86	1.82	81770	Rpl37	NM_031106
10796991	5.32	6.27	0.95	1.93	290963	Npepo	NM_001012346
10797469	5.11	6.00	0.89	1.85	25461	Hrh2	NM_012965
10798499	7.02	8.00	0.98	1.98	64627	Hist1h4b	NM_022686
10800426	3.36	4.23	0.88	1.84	24856	Ttr	NM_012681
10801821	4.50	5.35	0.85	1.80	116685	Lmnb1	NM_053905
10802128	8.53	9.64	1.11	2.17	124323	Rps23	NM_078617
10803025	4.32	5.58	1.25	2.38	361354	Fbxo15	NM_001108436
10805996	4.79	5.89	1.10	2.14	64364	Pllp	NM_022533
10806210	2.61	3.69	1.07	2.10	29225	Es22	NM_031565
10807044	4.69	5.95	1.26	2.39	688429	Arhgap10	NM_001109501
10807514	6.79	7.90	1.12	2.17	65139	Rps12	NM_031709
10807542	2.52	3.77	1.25	2.38	83502	Cdh1	NM_031334
10808107	3.72	4.66	0.93	1.91	498954	RGD1563834	XR_085682
10809402	7.21	8.71	1.50	2.83	117038	Mt3	NM_053968
10810299	7.25	8.21	0.96	1.94	361385	Ndufb7	NM_001108442
10812416	8.60	9.62	1.02	2.03	124323	Rps23	NM_078617
10812899	9.01	10.01	1.00	1.99	360572	Rpl23a	NM_001108283
10813330	8.98	9.94	0.96	1.94	81770	Rpl37	NM_031106
10813951	5.20	6.16	0.95	1.94	619558	Fam134b	ENSRNOT00000014423
10814540	6.08	7.19	1.11	2.16	310269	RGD1307225	NM_001107663
10816675	2.94	3.80	0.85	1.81	24651	Pklr	NM_012624
10821695	7.01	7.94	0.93	1.91	117053	Rps15a	NM_053982
10822005	4.65	5.54	0.88	1.85	78961	Golph3	NM_023977
10822929	2.77	3.67	0.89	1.86	207120	Nudt6	NM_181363
10824689	5.30	6.19	0.88	1.84	295214	S100a1	NM_001007636
10825236	9.55	10.49	0.94	1.92	689284	Rpl38	NM_001077592
10825238	1.68	2.60	0.92	1.89	365885	Olr390	NM_001000558
10825768	2.68	3.69	1.00	2.00	310771	Chi3l4	XM_001069770
10828433	7.49	8.53	1.04	2.06	294282	Rps18	NM_213557
10828737	7.31	8.18	0.86	1.82	81729	Rpl10a	NM_031065
10830624	6.65	7.54	0.89	1.85	25145	Cd24	NM_012752
10832847	3.57	4.44	0.87	1.83	309902	Tet1	NM_001107643

10833564	4.24	5.36	1.13	2.19	499454	RGD1560095	ENSRNOT00000040584
10834310	7.11	8.00	0.89	1.85	296570	Edf1	NM_001106557
10839400	8.99	9.86	0.86	1.82	28298	Rpl32	NM_013226
10839443	3.88	4.95	1.07	2.11	65192	Slc27a2	NM_031736
10839876	6.77	7.92	1.15	2.23	65139	Rps12	NM_031709
10840783	3.65	4.60	0.95	1.93	311536	RGD1304644	BC099097
10841484	5.64	6.74	1.11	2.15	679572	Romo1	ENSRNOT00000026856
10842436	8.85	9.73	0.89	1.85	689284	Rpl38	NM_001077592
10842667	4.55	5.43	0.88	1.84	64307	Rpl24	NM_022515
10842805	8.48	9.72	1.24	2.37	81775	Rps21	NM_031111
10842971	5.31	6.30	0.99	1.99	362288	Rtel1	ENSRNOT00000055030
10844327	6.85	7.93	1.08	2.12	499780	RGD1561113	ENSRNOT00000034930
10844946	7.17	8.33	1.15	2.23	29286	Rps17	NM_017152
10851386	8.32	9.31	0.99	1.98	81765	Rpl13	NM_031101
10851650	3.10	5.10	2.00	4.01	296369	Tnnc2	NM_001037351
10853871	2.28	3.49	1.21	2.31	24255	Cftr	NM_031506
10854386	2.69	3.57	0.88	1.84	296968	RGD1308226	NM_001106589
10857231	3.78	4.87	1.09	2.12	685999	Uroc1	ENSRNOT00000038251
10861676	9.43	10.31	0.89	1.85	689284	Rpl38	NM_001077592
10863218	4.07	5.08	1.01	2.01	83730	Vamp8	NM_031827
10863664	5.00	5.91	0.91	1.88	312504	RGD1566130	NM_001107871
10863679	5.96	7.29	1.33	2.52	114020	Cml5	NM_080884
10864121	4.38	5.53	1.16	2.23	171571	Grip2	NM_138535
10864425	5.51	6.40	0.89	1.85	297481	Eif4e3	NM_001106612
10864769	9.59	10.54	0.95	1.93	28298	Rpl32	NM_013226
10865630	3.83	4.84	1.00	2.01	362438	Ncapd2	ENSRNOT00000025889
10866505	8.90	9.85	0.95	1.93	690830	LOC690830	XM_001075809
10868673	4.81	5.82	1.01	2.01	680021	Grhpr	NM_001113754
10870043	6.60	7.45	0.85	1.80	294282	Rps18	NM_213557
10871071	2.78	3.71	0.93	1.91	313507	Tal1	NM_001107958
10871169	7.97	8.92	0.95	1.93	366448	Uqcrh	NM_001009480
10872015	3.84	4.99	1.16	2.23	362597	Fam176b	BC167091
10872093	7.21	8.08	0.88	1.84	57809	Rpl35a	NM_021264
10872139	6.41	7.28	0.87	1.83	286923	Dlgap3	NM_173138
10873322	10.01	10.92	0.90	1.87	287005	Camk2n1	NM_173337
10874691	6.17	7.06	0.88	1.85	117053	Rps15a	NM_053982

10875089	4.87	5.73	0.85	1.81	362472	Cspp1	ENSRNOT00000008480
10876652	3.62	4.71	1.09	2.12	362515	Anks6	NM_001015028
10877372	2.63	4.04	1.41	2.65	298107	Mup5	AB039828
10878428	9.57	10.45	0.87	1.83	28298	Rpl32	NM_013226
10878910	10.00	11.11	1.11	2.16	27139	Rps26	NM_013224
10878954	8.19	9.04	0.85	1.80	65136	Rps8	NM_031706
10880710	3.96	5.00	1.04	2.06	313633	Ephb2	NM_001127319
10881596	4.05	4.98	0.93	1.90	313705	Ptchd2	NM_001107992
10882221	5.23	6.10	0.86	1.82	680723	Tmem88b	NM_001109426
10886036	5.18	6.03	0.86	1.81	116674	Jdp2	NM_053894
10891400	5.47	6.44	0.98	1.97	64629	Porf1	ENSRNOT00000005162
10891679	3.84	4.84	1.00	2.00	314386	Gpr68	NM_001108049
10892702	9.55	10.45	0.90	1.87	28298	Rpl32	NM_013226
10892937	5.93	6.90	0.97	1.95	29287	Rps19	NM_001037346
10894055	4.90	5.87	0.97	1.96	314612	Shc2	NM_001108065
10894681	3.46	4.72	1.25	2.39	24616	Pah	NM_012619
10894810	6.69	7.96	1.27	2.41	314721	Anks1b	ENSRNOT00000064242
10894812	9.54	10.68	1.13	2.19	314721	Anks1b	ENSRNOT00000064242
10894814	8.48	9.80	1.32	2.49	314721	Anks1b	ENSRNOT00000064242
10897450	4.97	5.85	0.88	1.84	300317	Kctd17	NM_001134529
10899465	4.89	6.07	1.18	2.26	25641	Igfbp6	NM_013104
10899824	8.93	9.93	1.00	2.00	124440	Rpl41	NM_139083
10899868	9.28	10.32	1.04	2.05	27139	Rps26	NM_013224
10900533	7.47	8.59	1.12	2.17	29285	Rps15	NM_017151
10900618	7.94	8.86	0.92	1.89	29328	Gpx4	NM_017165
10901178	8.62	9.60	0.98	1.97	691531	Rps28	NM_001105730
10902047	2.67	4.50	1.83	3.56	299757	Nts	NM_001102381
10903583	6.68	7.68	1.01	2.01	81763	Rpl5	NM_031099
10904227	5.88	6.86	0.98	1.98	29287	Rps19	NM_001037346
10905277	6.56	7.47	0.91	1.88	25269	Pvalb	NM_022499
10905558	6.83	7.82	0.99	1.98	287417	Rpl26	NM_001105788
10907297	3.76	4.65	0.89	1.85	300236	Smagp	NM_182817
10907324	4.08	4.95	0.87	1.83	683264	Galnt6	NM_001172063
10908347	4.44	5.68	1.24	2.36	25638	Pde4a	NM_013101

10909002	3.39	4.80	1.41	2.67	619560	Rup2	NM_001034950
10909009	2.12	3.94	1.83	3.55	619560	Rup2	AF368860
10909758	8.33	9.51	1.19	2.28	81775	Rps21	NM_031111
10909892	6.43	7.39	0.96	1.95	25420	Cryab	NM_012935
10910458	9.55	10.49	0.94	1.92	689284	Rpl38	NM_001077592
10914308	9.02	10.12	1.10	2.14	29236	Rpsa	NM_017138
10915345	5.30	6.22	0.93	1.90	313783	Olfm2	NM_001015017
10918055	3.75	4.67	0.91	1.88	315741	Paqr5	NM_001014092
10918776	7.03	7.90	0.87	1.82	681849	LOC681849	ENSRNOT00000067627
10920967	2.90	3.76	0.86	1.81	363165	Csrnp1	NM_001108786
10921036	3.59	5.10	1.50	2.83	363168	Lyzl4	ENSRNOT00000026173
10921274	7.90	8.82	0.92	1.89	316164	Satb1	NM_001012129
10922268	4.54	5.66	1.12	2.18	170843	Khdrbs2	NM_133318
10924217	9.88	10.80	0.91	1.89	363248	Rpl37a	NM_001108801
10926386	4.45	5.39	0.94	1.93	681367	LOC681367	XM_001061427
10926683	5.93	6.98	1.05	2.07	301265	Pla2g7	NM_001009353
10927281	6.87	7.73	0.86	1.81	29284	Rps14	NM_022672
10927372	3.86	4.98	1.12	2.17	367251	Ankrd39	NM_001135014
10930091	6.64	7.55	0.91	1.88	316643	Chchd2	NM_001015019
10930762	1.98	3.22	1.24	2.36	171521	Cyp2c13	NM_138514
10933957	9.74	10.61	0.87	1.82	28298	Rpl32	NM_013226
10934500	9.55	10.53	0.99	1.98	27139	Rps26	NM_013224
10937356	9.22	10.26	1.05	2.07	27139	Rps26	NM_013224
10938962	8.59	9.64	1.04	2.06	81775	Rps21	NM_031111
10940358	3.97	5.10	1.13	2.19	293862	Fam50a	NM_001170573

Table S2B: List of genes downregulated in brain

Probe Set ID	Control Brain	Sleep Deprived Brain	Log Fold Change	Fold Change	Entrez ID	Gene Symbol	Accession
10702201	5.59	4.73	-0.86	0.55	361458	Akap7	NM_001001801
10702626	6.12	4.91	-1.21	0.43	361472	Mthfd1l	NM_001108462
10702991	4.68	3.67	-1.02	0.49	25153	Mas1	NM_012757
10705825	5.51	4.65	-0.86	0.55	246264	Zfp382	NM_144749

10708616	7.21	6.08	-1.13	0.46	293112	Crebzf	NM_001106279
10709020	9.84	8.79	-1.05	0.48	685076	Pgm2l1	NM_001109454
10709844	5.46	4.42	-1.04	0.49	308937	Wee1	NM_001012742
10715149	4.35	3.30	-1.05	0.48	294072	Tmem20	ENSRNOT00000044895
10715519	5.67	4.80	-0.87	0.55	293939	Erlin1	NM_001106353
10716026	6.12	5.05	-1.07	0.48	294043	Sorcs3	NM_001106367
10717053	3.43	2.38	-1.05	0.48	308717	Slc35d3	NM_001107522
10717069	6.29	5.25	-1.04	0.49	140929	Pde7b	NM_080894
10717325	4.44	3.51	-0.94	0.52	308028	Slc2a12	NM_001107451
10719134	4.63	3.58	-1.05	0.48	499083	Zscan18	ENSRNOT00000036880
10720126	3.49	2.62	-0.87	0.55	687333	LOC687333	ENSRNOT00000025586
10722218	4.11	3.23	-0.88	0.54	404658	Mrgprb4	ENSRNOT00000045792
10722532	5.36	4.09	-1.27	0.41	25302	Chrna7	NM_012832
10723351	4.35	3.48	-0.87	0.55	116996	Il16	NM_001105749
10724785	6.69	5.64	-1.05	0.48	361625	Nrip3	NM_001108498
10725778	5.06	4.18	-0.88	0.54	113900	Nupr1	NM_053611
10726999	4.57	3.28	-1.29	0.41	24483	Igf2	NM_031511
10727717	6.79	5.68	-1.11	0.46	266734	Npas4	NM_153626
10729314	9.46	8.61	-0.85	0.55	83585	Gda	NM_031776
10730206	7.14	6.28	-0.85	0.55	65047	Slit1	NM_022953
10734045	6.24	5.08	-1.16	0.45	287371	Lrrc48	NM_001013857
10734422	5.23	4.36	-0.88	0.54	117251	Dnah9	ENSRNOT00000005583
10739351	4.86	2.66	-2.20	0.22	29719	Kcnj16	NM_053314
10739984	4.91	4.02	-0.89	0.54	303735	Rnf213	ENSRNOT00000004904
10741907	6.97	6.01	-0.97	0.51	25498	Npm1	NM_012992
10743715	4.80	3.18	-1.62	0.32	497926	Shisa6	ENSRNOT00000057509
10744081	5.82	4.33	-1.49	0.36	360546	Efnb3	NM_001100980
10748498	5.07	3.95	-1.12	0.46	29481	Rgs9	NM_019224
10750551	7.40	6.53	-0.88	0.54	29202	Epha6	ENSRNOT00000047310

10750928	7.54	6.59	-0.95	0.52	303963	Dzip3	ENSRNOT00000002678
10751237	4.51	3.29	-1.22	0.43	498085	RGD1563835	ENSRNOT00000042756
10751700	7.89	7.00	-0.89	0.54	192361	Ppp1r2	NM_138823
10752576	5.49	4.49	-0.99	0.50	287925	Pkp2	NM_001100499
10752738	7.18	6.32	-0.87	0.55	29734	Hspa13	NM_019271
10753269	6.49	5.45	-1.03	0.49	25743	Kcnj6	NM_013192
10758020	7.30	6.33	-0.98	0.51	288620	Cct6a	NM_001033684
10762426	5.93	4.74	-1.20	0.44	117271	Hrk	NM_057130
10763421	3.92	2.91	-1.00	0.50	297865	Dsel	ENSRNOT00000043890
10763933	7.54	6.32	-1.22	0.43	289020	Lrrn2	NM_001177368
10764139	6.93	6.05	-0.88	0.54	304807	Rabif	NM_001007678
10764460	6.98	5.95	-1.04	0.49	304827	Kcnt2	NM_198762
10764551	5.85	4.97	-0.87	0.55	29527	Ptgs2	NM_017232
10764837	7.03	6.07	-0.97	0.51	304881	Tor1aip2	NM_001165896
10765044	4.73	3.84	-0.88	0.54	289150	Cenpl	NM_001033061
10765413	5.21	4.24	-0.97	0.51	83574	Rxrg	NM_031765
10766953	5.16	4.03	-1.13	0.46	24224	Bcl2	NM_016993
10768269	6.55	5.35	-1.20	0.44	155012	Cfh	NM_130409
10768357	4.45	2.88	-1.57	0.34	289076	Rgs18	NM_001047084
10769177	5.66	4.34	-1.31	0.40	684623	LOC684623	ENSRNOT00000003659
10770161	5.12	4.08	-1.03	0.49	289270	Pld5	ENSRNOT00000005332
10772534	8.47	7.28	-1.20	0.44	289606	Gabra2	NM_001135779
10774267	7.13	5.93	-1.20	0.44	689106	Vstm2a	ENSRNOT00000006897
10774432	4.50	3.42	-1.09	0.47	690096	LOC690096	ENSRNOT00000012255
10775283	4.45	3.15	-1.31	0.40	360910	Abcg3l2	NM_001014133
10776437	5.81	4.92	-0.89	0.54	64030	Kit	NM_022264
10777011	6.24	5.09	-1.15	0.45	289657	Tbc1d19	NM_001106008
10777137	5.84	4.39	-1.45	0.37	360272	Slit2	NM_022632
10778179	5.00	3.71	-1.29	0.41	114107	Kremen1	NM_053649
10781467	6.53	4.79	-1.73	0.30	29595	Htr2a	NM_017254
10781496	6.25	5.32	-0.94	0.52	306071	Lcp1	NM_001012044

10781745	6.67	5.81	-0.86	0.55	306055	Pcdh17	NM_001107279
10781787	9.58	8.69	-0.88	0.54	29150	Matr3	NM_019149
10782187	4.94	3.22	-1.72	0.30	498564	Itgbl1	NM_001017505
10782590	8.34	7.36	-0.98	0.51	66030	Synpr	NM_023974
10785724	8.09	7.04	-1.05	0.48	50672	Ednrb	NM_017333
10788542	3.41	2.12	-1.29	0.41	306511	RGD1561928	ENSRNOT00000042609
10788889	4.27	3.20	-1.07	0.48	306564	Htra4	NM_001107321
10791000	5.71	4.14	-1.56	0.34	290655	Crlf1	NM_001106074
10791250	5.42	3.71	-1.71	0.31	24539	Lpl	NM_012598
10791950	6.61	5.76	-0.85	0.55	364601	Efha2	ENSRNOT00000017025
10793429	4.53	3.42	-1.11	0.46	290952	RGD1563615	ENSRNOT00000046525
10793433	3.75	2.74	-1.01	0.50	290952	RGD1563615	ENSRNOT00000046525
10793446	4.99	3.56	-1.44	0.37	290952	RGD1563615	ENSRNOT00000046525
10794195	5.51	3.70	-1.81	0.28	24316	Drd1a	NM_012546
10796507	5.20	4.10	-1.10	0.47	291328	Slc39a12	NM_001106124
10796751	7.76	6.39	-1.37	0.39	24380	Gad2	NM_012563
10797648	6.68	5.41	-1.27	0.41	291015	Ogn	NM_001106103
10797657	3.29	2.36	-0.93	0.52	83717	Omd	NM_031817
10798119	4.41	3.26	-1.15	0.45	291084	Nqo2	NM_001004214
10798390	6.64	5.69	-0.94	0.52	306934	Fam65b	NM_001014009
10798479	3.67	2.59	-1.08	0.47	306966	Znf184	NM_001100573
10798856	6.86	5.93	-0.93	0.52	361258	Cul2	NM_001108417
10799733	7.92	6.65	-1.27	0.41	680404	C1ql3	NM_001109403
10800140	7.94	5.06	-2.88	0.14	291794	Snrpd1	NM_001106163
10801174	4.59	3.57	-1.02	0.49	291654	Pcdhb5	NM_001114602
10802391	5.47	3.56	-1.90	0.27	24611	Gnal	ENSRNOT00000025172
10803037	8.88	8.01	-0.87	0.55	307206	Neto1	NM_001107371
10803394	8.19	7.23	-0.96	0.51	65196	B4galt6	NM_031740

10803474	6.68	5.83	-0.85	0.55	307553	Nol4	NM_001107401
10804714	5.87	4.63	-1.24	0.42	307396	Pcyox1l	NM_001134542
10805591	3.35	2.35	-1.00	0.50	498895	RGD1559751	ENSRNOT00000058177
10806492	6.78	5.85	-0.93	0.53	25479	Vps35	ENSRNOT00000024020
10806864	7.13	6.15	-0.98	0.51	364981	Scoc	NM_001013235
10807632	7.40	6.41	-0.99	0.50	80773	Cyb5b	NM_030586
10808603	6.78	5.78	-1.00	0.50	361433	Cpne7	NM_001108454
10810736	5.74	4.63	-1.11	0.46	24530	Lcat	NM_017024
10810964	4.23	3.14	-1.10	0.47	117059	Calb2	NM_053988
10812879	6.16	4.36	-1.80	0.29	24473	Htr1a	NM_012585
10813249	7.21	5.41	-1.81	0.29	310358	RGD1308448	NM_001107671
10813563	4.73	3.80	-0.93	0.52	294789	Ranbp3l	ENSRNOT00000050942
10813817	4.59	3.27	-1.32	0.40	29163	Cdh9	NM_001168630
10813872	6.57	5.69	-0.88	0.54	310174	Cdh18	NM_001107656
10814396	5.37	3.50	-1.87	0.27	365748	Bhlhe22	NM_001108940
10815655	4.41	2.97	-1.44	0.37	66024	Npy2r	NM_023968
10815679	3.90	3.01	-0.88	0.54	24590	Mme	NM_012608
10817419	5.45	4.46	-0.98	0.51	29175	Ctsk	NM_031560
10820300	4.77	3.74	-1.03	0.49	309995	Xrcc4	NM_001006999
10820586	5.24	4.29	-0.95	0.52	25439	F2r	NM_012950
10820613	5.31	3.72	-1.59	0.33	29643	Sv2c	NM_031593
10820693	8.21	7.30	-0.90	0.53	294673	Hexb	NM_001011946
10822107	4.90	3.99	-0.91	0.53	310190	Fam105a	BC107649
10822386	7.64	6.64	-1.00	0.50	114906	Pkia	NM_053772
10823303	9.93	8.96	-0.97	0.51	81531	Pfn2	NM_030873
10823412	3.45	2.46	-0.99	0.50	192251	Gpr149	NM_138891
10823937	4.41	2.97	-1.44	0.37	66024	Npy2r	NM_023968
10824041	6.17	5.32	-0.85	0.55	60382	Arfip1	NM_021763
10824422	5.29	4.42	-0.87	0.55	310641	Trim46	NM_001107691
10825495	5.36	4.46	-0.89	0.54	310732	Slc22a15	NM_001107707
10825915	3.67	2.74	-0.93	0.52	24424	Gstm2	NM_177426
10826148	5.97	4.92	-1.05	0.48	295382	Ntng1	NM_001106465
10826261	7.18	5.74	-1.44	0.37	64443	Gpr88	NM_031696

10826392	8.58	7.35	-1.24	0.42	295401	Lppr4	NM_001001508
10827231	5.97	4.85	-1.12	0.46	83476	Cyr61	NM_031327
10828344	5.32	3.47	-1.85	0.28	294269	RT1-Da	NM_001008847
10832577	6.25	4.52	-1.73	0.30	25369	Adora2a	NM_053294
10836638	7.55	6.68	-0.87	0.55	311114	Klhl23	NM_001134504
10838039	7.57	6.37	-1.20	0.44	295934	Chst1	NM_001011955
10838255	3.59	2.70	-0.89	0.54	691083	LOC691083	NM_001144862
10838326	6.20	5.21	-0.99	0.50	25469	Kcna4	NM_012971
10839726	5.00	4.03	-0.97	0.51	64300	Mthfd1	NM_022508
10842677	4.91	4.01	-0.91	0.53	100328908	RGD1565819	NM_001171096
10844082	5.63	4.62	-1.01	0.50	311836	Ntng2	NM_001107825
10844275	4.36	3.20	-1.16	0.45	311861	Fibcd1	NM_001107829
10844801	6.59	5.72	-0.86	0.55	366029	Zbtb6	NM_001108953
10844949	5.19	4.15	-1.04	0.49	311926	Lrp1b	NM_001107843
10844960	6.15	5.29	-0.87	0.55	311926	Lrp1b	NM_001107843
10844962	5.43	4.30	-1.13	0.46	311926	Lrp1b	NM_001107843
10844968	4.90	3.89	-1.01	0.50	311926	Lrp1b	NM_001107843
10845416	5.96	4.84	-1.12	0.46	245921	Acvr1c	NM_139090
10845809	5.94	5.07	-0.87	0.55	497770	Scn3a	NM_013119
10846685	5.19	4.26	-0.94	0.52	29458	Neurod1	NM_019218
10847758	6.20	5.22	-0.99	0.51	366140	Fjx1	NM_001108955
10849857	4.89	3.79	-1.10	0.47	29190	Pdyn	NM_019374
10850543	8.11	7.00	-1.11	0.46	499903	Napb	ENSRNOT00000006329
10850929	7.15	6.27	-0.88	0.54	363469	Sms	NM_001033899
10852953	5.77	4.70	-1.06	0.48	499975	Fam126a	ENSRNOT00000014023
10853683	3.73	2.81	-0.92	0.53	24806	Tac1	NM_012666
10853842	8.47	7.59	-0.88	0.54	493810	Capza2	NM_001009180
10855549	6.05	3.66	-2.39	0.19	297096	Snx10	NM_001013085
10856472	8.08	7.16	-0.92	0.53	679668	LRRTM1	NM_001109374

10858165	7.87	6.97	-0.89	0.54	24772	Cxcl12	NM_001033883
10858707	3.03	1.99	-1.04	0.49	297594	Cdca3	NM_001007648
10859342	5.85	4.42	-1.42	0.37	50677	Ptpro	NM_017336
10860481	6.06	5.20	-0.86	0.55	29751	Sema3a	NM_017310
10860535	7.03	6.18	-0.85	0.56	688590	Rundc3b	NM_001047116
10860623	5.42	4.54	-0.89	0.54	85261	Mterf	NM_053499
10861038	6.66	5.72	-0.94	0.52	500032	Thsd7a	ENSRNOT00000052063
10861066	7.76	6.87	-0.88	0.54	312132	Tmem106b	NM_001004267
10862527	5.66	4.79	-0.87	0.55	155183	Skap2	NM_130413
10863615	7.24	6.38	-0.85	0.55	500233	Exoc6b	NM_001109246
10863688	3.64	2.68	-0.96	0.51	500237	Cml2	NM_001173449
10866576	9.42	8.49	-0.93	0.52	497798	RGD1561357	ENSRNOT00000010623
10866850	7.44	6.51	-0.93	0.53	24525	Kras	NM_031515
10867060	4.81	3.91	-0.90	0.54	500377	Tuba8	NM_001024339
10867497	7.68	6.66	-1.01	0.50	500400	Fam110b	BC091289
10867731	6.97	5.82	-1.15	0.45	83839	Calb1	NM_031984
10868186	7.18	6.16	-1.01	0.50	25248	Cnr1	NM_012784
10868289	8.60	7.66	-0.94	0.52	65028	Dnaja1	NM_022934
10869010	6.40	5.11	-1.29	0.41	313219	Zfp189	NM_001107930
10869094	5.75	4.90	-0.86	0.55	313211	Nipsnap3b	NM_001009422
10869253	7.81	6.88	-0.93	0.52	83626	Ugcg	NM_031795
10870733	5.04	4.15	-0.89	0.54	298385	Magoh	NM_001100536
10874193	7.78	6.83	-0.95	0.52	313729	Errfi1	NM_001014071
10875256	7.13	6.01	-1.12	0.46	312946	Tmem68	NM_001107903
10875300	6.07	4.18	-1.89	0.27	29237	Penk	NM_017139
10875532	6.43	5.55	-0.87	0.55	54705	Pdp1	NM_019372
10883801	7.18	5.38	-1.80	0.29	50871	Hpcal1	NM_017356
10884215	3.32	2.38	-0.94	0.52	85489	Twist1	NM_053530
10884309	8.50	7.34	-1.16	0.45	54248	Dgkb	NM_019304
10884811	6.93	5.74	-1.19	0.44	314164	Lrfn5	NM_001108024
10886031	5.13	4.18	-0.96	0.52	314322	Fos	NM_022197
10889575	6.65	5.37	-1.28	0.41	298944	Gpr22	NM_001106722
10890229	5.12	4.22	-0.90	0.54	85261	Mterf	NM_053499

10891402	4.94	3.37	-1.57	0.34	65162	Dio2	NM_031720
10893661	8.32	7.00	-1.32	0.40	58979	Gng7	NM_024138
10895152	6.37	5.29	-1.08	0.47	60427	Kitlg	NM_021843
10895241	3.96	2.79	-1.17	0.45	299756	Mgat4c	NM_0011358 14
10895499	4.22	2.85	-1.37	0.39	171048	Tspan8	NM_133526
10895508	5.30	4.14	-1.16	0.45	94202	Ptprr	NM_053594
10896541	9.05	8.18	-0.87	0.55	81526	Nov	NM_030868
10899552	5.39	4.30	-1.09	0.47	24790	Sp1	NM_012655
10899676	9.08	8.20	-0.88	0.54	29691	Pde1b	NM_022710
10901409	6.98	6.13	-0.85	0.55	314694	Chst11	NM_0011080 79
10902080	4.56	3.70	-0.86	0.55	299762	Tmtc2	XM_0010807 32
10902843	6.50	5.65	-0.85	0.56	171329	Usp15	NM_145184
10903501	6.45	5.15	-1.30	0.41	314941	Lrp12	NM_0011348 83
10903651	4.58	2.92	-1.66	0.32	314942	LOC314 942	ENSRNOT00 000057108
10903653	4.22	2.93	-1.29	0.41	314942	LOC314 942	ENSRNOT00 000057108
10908990	4.79	3.89	-0.90	0.54	315554	Pus3	NM_0011081 34
10909328	8.21	7.06	-1.15	0.45	245956	Scn3b	NM_139097
10909849	4.42	3.21	-1.21	0.43	24318	Drd2	NM_012547
10911380	4.44	3.55	-0.89	0.54	116676	Aldh1a2	NM_053896
10912054	4.04	2.94	-1.09	0.47	363111	Ripply2	ENSRNOT00 000013285
10912614	3.65	2.32	-1.33	0.40	24546	Slco2a1	NM_022667
10912718	7.27	5.42	-1.85	0.28	367160	Cpne4	NM_0011090 03
10914935	7.80	6.49	-1.31	0.40	363015	RGD131 0444	XM_0027298 71
10916379	4.01	2.91	-1.10	0.47	300608	RGD131 1744	ENSRNOT00 000066669
10916697	5.45	4.25	-1.20	0.44	315599	Nlrx1	NM_0010250 10
10918955	4.55	3.60	-0.96	0.51	246776	Filip1	NM_145682
10918979	6.52	5.08	-1.44	0.37	25075	Htr1b	NM_022225
10919694	5.49	4.62	-0.87	0.55	300967	Tmem10 8	ENSRNOT00 000014519
10921208	3.08	2.07	-1.01	0.50	85426	Slc5a7	NM_053521
10922895	4.52	2.96	-1.56	0.34	24785	Slc9a4	NM_173098
10922909	4.31	3.35	-0.96	0.51	24783	Slc9a2	NM_012653
10923338	5.36	4.05	-1.31	0.40	301416	Coq10b	NM_0010096 71
10923782	9.44	8.34	-1.10	0.47	286928	Abi2	NM_173143
10923866	6.30	5.35	-0.95	0.52	501153	Zdbf2	ENSRNOT00 000016038

10926740	6.98	5.83	-1.15	0.45	316256	Tnfrsf21	NM_001108207
10927903	6.89	5.83	-1.07	0.48	363228	Tmeff2	NM_001108795
10928056	8.26	7.29	-0.97	0.51	316400	Pgap1	NM_201990
10928522	7.81	6.92	-0.89	0.54	316444	Mdh1b	ENSRNOT00000017207
10928837	6.77	5.43	-1.34	0.40	25285	Igfbp5	NM_012817
10929263	7.98	6.41	-1.57	0.34	24765	Scg2	NM_022669
10929482	8.47	6.87	-1.60	0.33	316573	Dner	BC086329
10929536	4.82	3.62	-1.21	0.43	316578	Slc16a14	NM_001108229
10931669	4.80	3.93	-0.87	0.55	288979	C1ql2	NM_001105949
10932066	7.51	6.54	-0.97	0.51	313445	Klhl13	ENSRNOT00000067114
10932646	7.61	6.75	-0.86	0.55	360785	Ap1s1	BC168682
10932795	7.72	6.72	-1.00	0.50	113976	Acsl4	NM_053623
10933393	4.16	2.48	-1.68	0.31	24397	Glra2	NM_012568
10934270	4.56	3.59	-0.97	0.51	302421	Gdpd2	NM_001106944
10935038	4.09	3.17	-0.92	0.53	501624	Bex4	NM_001037554
10935418	3.16	1.96	-1.20	0.44	317589	Mst4	ENSRNOT00000010596
10935882	3.50	2.44	-1.07	0.48	293849	Zfp275	NM_001106343
10936360	6.95	5.55	-1.40	0.38	313436	Zcchc12	NM_001014065
10936742	5.07	4.21	-0.86	0.55	554353	Gpr34	NM_001024925
10937302	6.64	5.29	-1.35	0.39	25187	Htr2c	NM_012765
10937362	7.96	7.06	-0.91	0.53	29433	Pak3	NM_019210
10937479	7.81	6.90	-0.91	0.53	117043	RragB	NM_053972
10937660	6.97	6.03	-0.93	0.52	501550	Trappc2	NM_001024965
10937769	5.22	4.15	-1.07	0.48	302671	Ap1s2	NM_001127531
10938897	7.98	7.02	-0.96	0.51	246284	Atrx	NM_001105757
10939002	6.31	5.02	-1.29	0.41	363487	RGD1566265	NM_001134589
10939226	6.47	5.11	-1.35	0.39	317183	Pcdh19	NM_001169129
10939460	6.24	5.37	-0.88	0.54	367915	Rab9b	NM_001109018
10939764	4.76	3.07	-1.69	0.31	25236	Gpc3	NM_012774
10939837	7.92	6.97	-0.95	0.52	302864	Mmgt1	NM_001106970

Table S2C: List of genes upregulated in liver

Probe Set ID	Control Liver	Sleep Deprived Liver	Log Fold Change	Fold Change	Gene Symbol	Accession
10704284	9.50	10.74	1.24	2.36	Slc27a5	NM_024143
10707151	5.06	6.32	1.25	2.39	Tmem86a	NM_001135016
10708665	4.24	5.34	1.10	2.15	Rab30	NM_001015012
10712657	8.66	9.95	1.28	2.44	Cpt1a	NM_031559
10713974	4.99	5.86	0.88	1.83	Slc15a3	NM_139341
10714323	5.37	7.66	2.29	4.91	Aldh1a1	NM_022407
10714413	6.98	7.94	0.96	1.94	Klf9	NM_057211
10715364	9.40	10.92	1.51	2.86	Got1	NM_012571
10715416	4.57	6.75	2.18	4.54	Loxl4	NM_001107592
10717459	4.93	6.34	1.40	2.65	Tppp	NM_001108461
10723728	6.21	7.14	0.93	1.91	Aqp11	NM_173105
10726604	4.07	5.01	0.94	1.91	RGD1311186	NM_001106310
10726824	8.05	9.02	0.98	1.97	Slc25a22	NM_001014027
10728466	5.16	6.89	1.73	3.32	UST4r	NM_134379
10730031	3.13	5.51	2.39	5.23	Cyp2c13	ENSRNOT00000015801
10730599	4.87	7.61	2.74	6.70	Cyp17a1	NM_012753
10732068	7.27	8.13	0.86	1.82	Dci	NM_017306
10736863	3.74	4.62	0.88	1.84	Ccl4	NM_053858
10738399	8.91	10.70	1.79	3.45	G6pc	NM_013098
10738477	3.88	5.02	1.14	2.21	Arl4d	NM_001107052

10738576	7.84	8.76	0.92	1.90	Grn	NM_017113
10742645	4.90	6.28	1.37	2.59	Slc22a5	NM_019269
10744233	2.97	4.05	1.08	2.12	Tmem102	ENSRNOT00000020578
10744245	2.86	3.80	0.95	1.93	Nlgn2	NM_053992
10744460	4.84	5.85	1.02	2.02	Cxcl16	NM_001017478
10745345	3.42	4.50	1.08	2.12	Evi2a	ENSRNOT00000039261
10746139	7.90	8.90	1.00	2.00	Scpep1	NM_133383
10747262	3.15	4.12	0.97	1.96	Krt19	NM_199498
10749612	9.75	10.69	0.94	1.92	Actg1	NM_001127449
10750524	6.77	8.04	1.27	2.41	Mx2	NM_134350
10751295	4.15	5.52	1.38	2.60	Pla1a	NM_138882
10751352	8.17	9.78	1.61	3.05	Krt8	NM_199370
10751896	3.49	5.92	2.43	5.39	Cldn1	NM_031699
10754058	3.31	4.21	0.90	1.87	Ccdc52	NM_001008285
10755088	3.49	4.39	0.90	1.86	Rtp4	NM_001108321
10757082	4.05	4.99	0.94	1.91	Zfand2a	NM_001008363
10757632	4.16	5.44	1.28	2.43	Ccl24	NM_001013045
10757962	9.23	10.74	1.52	2.86	Asl	NM_021577
10759018	6.45	7.39	0.94	1.92	Mlec	NM_001013983
10762324	7.63	9.17	1.54	2.90	Sds	NM_053962
10762740	4.06	5.27	1.21	2.31	Oasl	NM_001009681
10765090	10.14	11.25	1.12	2.17	Actg1	NM_001127449
10766923	5.66	7.02	1.36	2.57	Rnf152	NM_001106305

10767175	7.69	9.22	1.53	2.89	Insig2	NM_178091
10767290	6.12	7.58	1.46	2.75	Acmsd	NM_134372
10768998	3.90	4.80	0.90	1.87	Tor3a	NM_001009683
10769361	8.58	9.51	0.93	1.90	Fmo1	NM_012792
10769476	6.52	8.11	1.59	3.01	Atp1b1	NM_013113
10770710	3.53	4.74	1.21	2.31	Atf3	NM_012912
10771655	3.40	4.56	1.15	2.23	Cxcl10	NM_139089
10771660	4.50	6.57	2.06	4.17	Cxcl9	NM_145672
10771936	7.90	8.88	0.97	1.96	Ugt2a3	NM_001135869
10771998	6.48	7.47	0.99	1.99	RGD1559459	ENSRNOT00000065079
10772986	4.27	5.36	1.09	2.12	Ppargc1a	NM_031347
10773221	7.64	8.70	1.05	2.08	Slc2a9	ENSRNOT00000042200
10773773	8.07	8.92	0.86	1.81	Sec14l4	NM_001109090
10776954	5.68	6.55	0.86	1.82	Rell1	NM_001113776
10777918	7.04	7.88	0.85	1.80	Drg1	NM_001009685
10778620	4.72	6.18	1.46	2.75	Slc1a4	NM_198763
10779673	4.79	5.68	0.89	1.85	Lgals3	NM_031832
10781321	3.79	5.63	1.84	3.58	Tnfrsf10b	NM_001108873
10789857	5.38	6.28	0.90	1.87	Il17rb	NM_001107290
10790670	4.61	5.71	1.10	2.14	Klf2	NM_001007684
10791233	7.28	8.30	1.02	2.02	Atp6v1b2	NM_057213
10791552	3.60	5.41	1.82	3.52	Gpm6a	NM_178105
10794829	4.47	5.39	0.92	1.90	Tubb2a	NM_001109119

10796016	5.55	6.43	0.87	1.83	RGD1564865	NM_001164396
10796018	6.82	8.02	1.21	2.31	RGD1564865	NM_001164396
10796440	5.01	6.00	0.99	1.99	Pter	NM_022224
10796445	6.41	7.40	0.98	1.98	Vim	NM_031140
10797811	3.18	4.07	0.89	1.85	Cd83	NM_001108410
10800991	6.04	7.19	1.15	2.22	Pura	ENSRNOT00000025756
10802013	7.54	8.94	1.40	2.63	Cd74	NM_013069
10803653	9.91	10.90	0.99	1.99	Actg1	NM_001127449
10805731	4.58	5.43	0.86	1.81	Tk2	NM_001106166
10807464	5.89	6.92	1.02	2.03	Pla2g15	NM_001004277
10813214	9.80	10.77	0.97	1.96	Hmgcs1	NM_017268
10813628	2.51	3.97	1.47	2.76	Prlr	NM_012630
10813992	7.13	8.16	1.04	2.05	LOC683212	XR_086164
10815281	5.43	6.49	1.05	2.08	Ccrn4l	NM_138526
10815503	10.49	11.53	1.04	2.05	Tm4sf4	NM_053785
10817686	7.70	8.81	1.11	2.16	Fmo5	NM_144739
10818090	7.53	8.51	0.98	1.97	Slc16a1	NM_012716
10819024	5.60	6.51	0.90	1.87	Pla2g12a	NM_001108565
10819402	2.52	4.70	2.18	4.54	Adh6	NM_001012084
10819523	4.21	5.18	0.97	1.96	Gbp2	NM_133624
10819644	7.37	8.24	0.87	1.83	Ddah1	NM_022297
10819905	5.36	6.45	1.08	2.12	Ptger3	NM_012704

10820494	11.83	12.95	1.12	2.17	Bhmt	NM_030850
10821851	2.77	4.11	1.34	2.53	Il7r	NM_001106418
10825244	5.76	6.88	1.13	2.18	Chd1l	NM_001107704
10826691	6.12	7.06	0.94	1.92	Ap1ar	ENSRNOT00000055607
10827517	9.91	11.24	1.33	2.51	Cth	NM_017074
10828154	5.24	6.18	0.94	1.92	Hspa1b	NM_212504
10828344	7.29	8.92	1.63	3.10	RT1-Da	NM_001008847
10828351	4.88	6.01	1.14	2.20	RT1-Ba	NM_001008831
10829244	4.28	5.26	0.98	1.97	Pwp2	NM_001168653
10829346	3.57	4.46	0.89	1.85	Lrrc3	NM_145679
10830630	5.37	6.62	1.25	2.38	Rtn4ip1	NM_001107644
10831308	2.88	3.92	1.03	2.05	Slc44a4	NM_212541
10831567	5.17	6.46	1.29	2.45	RT1-Bb	NM_001004084
10831747	5.06	6.22	1.15	2.22	Wdr46	NM_212491
10832563	6.63	7.51	0.88	1.84	Gstt3	NM_001137643
10833617	6.33	7.55	1.22	2.33	RGD1561777	ENSRNOT00000035047
10833659	6.48	7.40	0.93	1.90	Amd1	NM_031011
10835703	3.86	5.00	1.14	2.20	RGD1564854	NM_001109341
10836019	9.10	10.25	1.15	2.21	Kynu	NM_053902
10836277	4.67	5.57	0.90	1.87	Gpd2	NM_012736
10837537	9.82	10.79	0.97	1.95	Actg1	NM_001127449
10838729	3.75	4.70	0.95	1.94	Rpusd2	NM_001135845

10839771	6.92	7.80	0.88	1.84	Slc20a1	NM_031148
10840085	6.82	7.71	0.90	1.86	Cds2	NM_053643
10841240	3.71	5.20	1.49	2.82	LOC690521	ENSRNOT00000042148
10844183	4.61	6.41	1.79	3.47	Crat	NM_001004085
10844801	5.23	6.17	0.94	1.92	Zbtb6	NM_001108953
10846604	4.28	5.20	0.91	1.88	LOC311134	ENSRNOT00000037015
10846781	3.94	4.86	0.93	1.90	Tfpi	NM_001177321
10849275	7.28	9.30	2.02	4.05	Slc28a2	NM_031664
10849279	6.12	7.06	0.94	1.92	Slc28a2	NM_031664
10850631	7.22	8.24	1.02	2.03	RGD1308874	NM_001034003
10850775	4.57	5.43	0.87	1.82	Trib3	NM_144755
10853229	4.81	5.94	1.13	2.18	Gnai1	NM_013145
10853300	5.46	6.66	1.21	2.31	Abcb1a	NM_133401
10853816	4.05	4.98	0.92	1.89	Cav1	NM_031556
10854548	11.18	12.13	0.95	1.93	Akr1d1	NM_138884
10855449	5.42	6.88	1.46	2.75	Gpnmb	NM_133298
10857314	4.72	5.89	1.17	2.24	Slc6a6	NM_017206
10858374	9.06	9.97	0.91	1.88	Slc6a13	NM_133623
10860076	4.59	5.44	0.86	1.81	Nos3	NM_021838
10860548	5.73	6.91	1.18	2.26	Crot	NM_031987
10861066	7.30	8.23	0.93	1.90	Tmem106b	NM_001004267
10861986	9.65	10.58	0.93	1.90	Insig1	NM_022392
10867329	2.75	4.05	1.30	2.46	Mybl1	NM_001106632

10870773	4.88	5.91	1.03	2.04	RGD1305274	NM_001106674
10873688	4.42	5.35	0.93	1.91	Arhgef19	NM_001108692
10875119	2.84	3.73	0.89	1.86	RGD1561849	NM_001109260
10877907	6.87	8.03	1.16	2.23	Adfp	NM_001007144
10878157	6.82	8.22	1.40	2.64	Cyp2j4	NM_023025
10878780	9.69	11.14	1.45	2.72	Cyp4a2	NM_001044770
10878787	9.61	10.94	1.33	2.52	Cyp4a3	NM_175760
10878938	4.91	6.33	1.41	2.66	Plk3	NM_022187
10879380	5.99	6.88	0.89	1.86	Ppcs	NM_001039010
10879516	8.47	9.96	1.48	2.80	Mfsd2	NM_001106683
10885851	5.03	6.36	1.33	2.52	Acot2	NM_138907
10886786	8.86	10.17	1.31	2.48	Hdmcp	NM_001001509
10887622	5.02	5.93	0.92	1.89	Crip	NM_001134933
10888196	5.82	6.74	0.92	1.90	Abcg5	NM_053754
10889728	4.60	5.53	0.92	1.90	Arl4a	NM_019186
10890206	6.12	7.25	1.13	2.18	Rpl10l	ENSRNOT00000047597
10893035	3.38	4.24	0.86	1.82	Timeless	NM_031340
10894100	6.59	7.60	1.01	2.01	Ppap2c	NM_139252
10894167	5.04	6.03	0.99	1.99	Cyp4f39	ENSRNOT00000007548
10897304	4.22	5.14	0.92	1.89	Gpr172b	NM_001109670
10897360	7.99	8.93	0.95	1.93	Gpt	NM_031039
10899713	9.07	10.03	0.96	1.95	Hsd17b6	NM_173305

10899764	3.43	4.46	1.03	2.04	Slc39a5	NM_001108728
10900905	2.69	3.56	0.87	1.83	Olr1088	ENSRNOT000000 12907
10901910	7.09	7.97	0.89	1.85	Amdhd1	ENSRNOT000000 07331
10907524	8.28	9.87	1.59	3.01	Krt8	NM_199370
10908108	3.81	4.75	0.93	1.91	RGD13094 10	NM_001134584
10909072	4.68	5.53	0.86	1.81	Esam	NM_001004245
10911145	2.94	5.64	2.70	6.51	Car12	NM_001080756
10911287	5.88	7.02	1.14	2.20	Anxa2	NM_019905
10913487	6.80	7.68	0.89	1.85	Shisa5	NM_001006989
10914411	7.53	9.59	2.06	4.16	Cyp8b1	NM_031241
10915018	3.66	4.66	1.00	2.00	Panx1	NM_199397
10917727	4.59	5.47	0.87	1.83	Neil1	NM_001025754
10918288	4.81	5.70	0.89	1.85	Plekho2	ENSRNOT000000 42633
10918364	4.58	5.71	1.12	2.18	LOC691658	ENSRNOT000000 57704
10918848	5.85	7.04	1.20	2.29	Slc17a5	NM_001009713
10919712	6.15	7.43	1.28	2.43	Ccrl1	ENSRNOT000000 15265
10921687	8.77	10.55	1.78	3.44	Gnmt	NM_017084
10922151	2.57	3.84	1.28	2.42	Gsta5	NM_001009920
10924223	3.91	5.73	1.82	3.52	Igfbp2	NM_013122
10928902	3.98	4.96	0.97	1.97	Tmbim1	NM_001007713
10930738	6.37	7.39	1.02	2.03	Oit3	NM_001001507
10931017	3.49	4.51	1.02	2.02	Sema6b	NM_053471
10933366	5.47	7.36	1.88	3.69	Rab9a	NM_053458

10933809	7.28	8.61	1.32	2.50	Tmem47	NM_001109317
10935177	7.17	8.06	0.89	1.85	Cldn2	NM_001106846
10937254	7.41	8.28	0.87	1.83	Pls3	NM_031084
10937570	7.11	7.96	0.85	1.80	LOC685431	XR_086366
10939310	6.42	7.45	1.03	2.04	Gla	NM_001108820

Table S2D: List of genes downregulated in liver

Probe Set ID	Control Liver	Sleep Deprived Liver	Log Fold Change	Fold Change	Entrez ID	Gene Symbol	Accession
10703272	4.73	3.83	-0.90	0.53	365122	RGD1561706	ENSRNOT00000059957
10705230	8.49	6.01	-2.48	0.18	361523	Cyp2b2	ENSRNOT00000028196
10708687	4.26	3.21	-1.05	0.48	499205	Fam181b	BC158703
10709844	4.60	3.54	-1.06	0.48	308937	Wee1	NM_001012742
10710647	5.18	4.30	-0.88	0.54	25023	Prkcb	NM_012713
10714254	4.64	3.51	-1.12	0.46	499330	LOC499330	NM_001024292
10717325	4.59	3.51	-1.08	0.47	308028	Slc2a12	NM_001107451
10720555	7.09	6.18	-0.91	0.53	688574	LOC688574	ENSRNOT00000043132
10721880	3.44	2.53	-0.91	0.53	308586	Sec1	NM_001135584
10724311	7.95	6.97	-0.98	0.51	24440	Hbb	NM_033234
10724319	7.21	6.06	-1.14	0.45	361619	MGC72973	NM_198776
10730310	9.14	7.44	-1.70	0.31	309375	Marveld1	NM_001107590
10730349	10.89	8.35	-2.54	0.17	246074	Scd1	NM_139192
10730830	5.16	4.15	-1.01	0.50	308760	Rccd1	ENSRNOT00000029471
10732652	9.55	8.54	-1.00	0.50	114856	Dusp1	NM_053769
10736875	10.19	8.75	-1.44	0.37	360228	LOC360228	NM_001003706

10741268	6.07	5.22	-0.85	0.55	302983	Mapk8ip3	NM_001100673
10741756	11.56	10.70	-0.85	0.55	25632	Hba-a2	NM_013096
10741778	11.16	10.29	-0.87	0.55	25632	Hba-a2	NM_013096
10742402	5.82	4.55	-1.27	0.41	114097	Ltc4s	NM_053639
10744766	5.02	4.08	-0.94	0.52	79251	Aspa	NM_024399
10745224	6.04	4.17	-1.87	0.27	65202	Slc13a2	NM_031746
10746209	6.84	5.70	-1.15	0.45	363667	Hlf	ENSRNOT00000055664
10749372	6.19	4.81	-1.38	0.38	89829	Socs3	NM_053565
10751931	7.55	6.03	-1.53	0.35	303836	Bcl6	NM_001107084
10753214	10.17	9.12	-1.06	0.48	266766	Rcan1	NM_153724
10756075	3.63	2.77	-0.87	0.55	501794	Vom2r60	ENSRNOT00000041244
10757442	4.32	3.43	-0.89	0.54	288585	Rabl5	NM_001011902
10759604	3.15	2.29	-0.86	0.55	498128	RGD1565374	NM_001134602
10760877	5.71	4.55	-1.16	0.45	288545	RGD1305455	NM_001024969
10761992	6.78	5.54	-1.25	0.42	29665	P2rx7	NM_019256
10762426	3.88	3.00	-0.88	0.54	117271	Hrk	NM_057130
10766835	8.30	7.35	-0.96	0.51	289392	Plxna2	NM_001105988
10767518	6.91	6.05	-0.87	0.55	363984	Ikbke	NM_001108854
10767539	6.35	5.27	-1.07	0.47	360840	Srgap2	NM_001134958
10768412	8.79	7.57	-1.22	0.43	289104	Prg4	NM_001105962
10772212	5.73	4.85	-0.88	0.54	364136	Aasdh	ENSRNOT00000002907
10775243	4.50	3.61	-0.89	0.54	363337	LOC363337	BC082068
10775260	4.56	3.26	-1.30	0.41	363337	LOC363337	BC082068
10775647	4.08	3.01	-1.07	0.48	289486	Fras1	ENSRNOT00000002814
10775731	4.31	3.28	-1.03	0.49	498335	Cxcl13	NM_001017496
10775900	8.58	6.83	-1.75	0.30	81503	Cxcl1	NM_030845
10776873	4.61	3.62	-0.99	0.50	305350	RGD1565119	ENSRNOT00000048106

10779093	6.24	5.36	-0.89	0.54	408223	Usp54	NM_001008863
10781273	5.02	3.13	-1.89	0.27	81801	Stc1	NM_031123
10781525	5.51	4.30	-1.20	0.43	113936	Cpb2	BC107447
10784980	7.32	6.11	-1.20	0.43	282843	Sorbs3	NM_001005762
10789442	9.65	8.50	-1.16	0.45	58935	Gas6	NM_057100
10789869	6.13	5.10	-1.03	0.49	29716	Cacna1d	NM_017298
10796831	4.16	3.28	-0.88	0.54	291345	RGD1561231	ENSRNOT00000047232
10797527	7.79	6.44	-1.35	0.39	291005	Gadd45g	NM_001077640
10797929	6.23	5.08	-1.15	0.45	306860	Gcnt2	NM_001001511
10798438	6.98	6.07	-0.91	0.53	24828	Hist1h2aa	ENSRNOT00000050526
10798943	5.58	4.61	-0.97	0.51	113938	Snurf	NM_130738
10798964	8.08	7.09	-0.99	0.50	85419	Lyst	NM_053518
10799888	5.28	4.15	-1.12	0.46	291367	RGD1564552	ENSRNOT00000050803
10800024	4.75	3.61	-1.13	0.46	291345	RGD1561231	ENSRNOT00000047232
10800919	6.57	5.45	-1.13	0.46	24330	Egr1	NM_012551
10801260	4.11	2.90	-1.21	0.43	680470	Pcdhgb6	ENSRNOT00000060466
10803194	5.52	4.65	-0.87	0.55	680014	Esco1	NM_001126299
10803692	7.16	5.96	-1.20	0.44	338475	Nrep	NM_178096
10804127	4.46	3.49	-0.97	0.51	25317	Fgf1	NM_012846
10804281	4.97	3.78	-1.19	0.44	24833	Spink3	NM_012674
10806191	4.74	3.79	-0.95	0.52	679726	LOC679726	ENSRNOT00000040473
10812734	5.61	4.76	-0.85	0.55	502503	Serf1	ENSRNOT00000024291
10813949	5.29	4.13	-1.16	0.45	619558	Fam134b	ENSRNOT00000014423
10814184	3.82	2.68	-1.14	0.45	310218	Car1	NM_001107660
10817142	4.31	3.43	-0.87	0.55	310586	RGD1566121	ENSRNOT00000035763
10817222	7.94	7.05	-0.89	0.54	499668	Lingo4	NM_001109189
10821698	5.01	4.10	-0.91	0.53	310132	Osmr	NM_001005384

10822242	11.78	10.22	-1.56	0.34	54232	Car3	NM_019292
10826216	6.60	5.68	-0.92	0.53	691538	Rnpc3	NM_0011008 10
10826723	8.96	7.91	-1.05	0.48	64017	Enpep	NM_022251
10826967	4.12	3.00	-1.11	0.46	365948	Bank1	XM_345295
10830223	9.15	8.29	-0.85	0.55	80841	Fabp7	NM_030832
10830270	5.06	3.41	-1.64	0.32	499453	RGD1565 550	ENSRNOT00 000044322
10830279	7.05	5.99	-1.07	0.48	499454	RGD1560 095	ENSRNOT00 000040584
10830854	7.92	6.70	-1.22	0.43	29168	Ubd	NM_053299
10832487	4.55	3.21	-1.34	0.39	690315	Derl3	NM_0011095 77
10832772	7.40	5.83	-1.58	0.34	309722	Rhobtb1	NM_0011076 22
10834335	3.76	2.76	-1.00	0.50	414138	Lcn6	NM_0010015 19
10834670	4.76	3.70	-1.07	0.48	311827	Adamtsl2	ENSRNOT00 000036995
10838443	2.88	1.99	-0.88	0.54	405231	Olr786	NM_0010009 16
10844331	10.33	5.05	-5.28	0.03	170496	Lcn2	NM_130741
10846652	5.59	4.71	-0.88	0.54	311137	Zfp385b	NM_0011077 36
10847957	6.36	4.55	-1.80	0.29	499847	Prrg4	NM_0011092 03
10857130	7.91	7.06	-0.86	0.55	29254	Mgll	NM_138502
10858408	6.78	4.64	-2.14	0.23	24153	A2m	NM_012488
10859671	4.66	3.81	-0.85	0.56	362464	LOC3624 64	ENSRNOT00 000055473
10860314	3.51	2.59	-0.92	0.53	286924	Gnat3	NM_173139
10861793	7.82	6.60	-1.21	0.43	503568	Slc13a4	NM_0010126 21
10863608	3.90	2.79	-1.11	0.46	312495	Cyp26b1	NM_181087
10863686	6.71	5.01	-1.70	0.31	64570	Nat8	NM_022635
10867497	4.65	3.55	-1.10	0.47	500400	Fam110b	BC091289
10867667	7.52	6.51	-1.01	0.50	117048	Cdh17	NM_053977
10869476	11.86	10.50	-1.36	0.39	24614	Orm1	NM_053288
10875567	5.33	4.35	-0.98	0.51	297903	Fam92a1	ENSRNOT00 000022249
10877372	7.29	5.84	-1.45	0.37	298107	Mup5	AB039828
10877755	8.66	7.71	-0.96	0.51	29227	Nfib	NM_031566
10881861	7.79	6.39	-1.40	0.38	78962	Per3	NM_023978

10887486	5.85	4.98	-0.86	0.55	362790	RGD1308350	ENSRNOT00000018225
10887492	5.87	4.70	-1.17	0.44	362790	RGD1308350	ENSRNOT00000018225
10887939	4.89	4.03	-0.85	0.55	313838	Cdc42ep3	NM_001048044
10890547	10.65	9.16	-1.48	0.36	299131	Dhrs7	NM_001013098
10894414	3.61	2.70	-0.91	0.53	300308	LOC300308	NM_001013952
10894525	5.94	5.07	-0.87	0.55	299691	Cry1	NM_198750
10895888	6.80	5.91	-0.89	0.54	362894	R3hdm2	NM_001130557
10895915	7.67	5.34	-2.33	0.20	362895	Stac3	NM_001130558
10903573	6.94	4.77	-2.17	0.22	500865	Golsyn	ENSRNOT00000005584
10905312	5.60	4.17	-1.43	0.37	171044	Sstr3	NM_133522
10906296	5.24	4.31	-0.92	0.53	500917	Odf3b	ENSRNOT00000055805
10909105	3.63	2.35	-1.28	0.41	406021	Olr1197	NM_001001120
10912076	4.87	3.87	-1.00	0.50	502642	RGD1563348	NM_001114939
10912908	7.43	6.46	-0.97	0.51	83681	Cish	NM_031804
10913043	4.46	3.54	-0.92	0.53	300999	Mst1r	NM_001106855
10917518	7.06	6.08	-0.98	0.51	315676	Dmxl2	ENSRNOT00000066197
10917617	6.35	4.53	-1.82	0.28	690919	Nrg4	ENSRNOT00000020332
10919010	4.85	3.93	-0.93	0.53	113990	Hmgn3	NM_001007020
10919262	5.53	4.56	-0.96	0.51	300888	Tmed3	NM_001004249
10920002	5.62	4.63	-0.99	0.50	315992	Dock3	NM_001108184
10920498	4.91	3.95	-0.96	0.51	316014	Nbeal2	ENSRNOT00000056130
10921141	7.34	6.47	-0.87	0.55	301085	Fyco1	NM_001106870
10922260	4.20	3.31	-0.89	0.54	316304	Lgsn	NM_181383
10922499	4.42	3.28	-1.14	0.45	679806	Fer1l5	ENSRNOT00000059806

10923432	10.86	9.46	-1.39	0.38	493909	Aox3	NM_0010085 27
10923474	7.39	6.11	-1.28	0.41	316424	Aox4	ENSRNOT00 000033068
10923476	11.44	10.02	-1.43	0.37	316424	Aox4	ENSRNOT00 000033068
10924245	3.36	2.31	-1.04	0.49	29385	Il8rb	NM_017183
10926651	7.75	6.32	-1.43	0.37	316249	Enpp5	NM_0010127 44
10926851	6.05	4.57	-1.48	0.36	301287	Pkhd1	ENSRNOT00 000060760
10928191	4.39	3.33	-1.06	0.48	501145	Satb2	NM_0011093 06
10928452	7.58	6.68	-0.91	0.53	363239	Raph1	NM_0011087 98
10929842	6.68	5.75	-0.93	0.53	63840	Per2	NM_031678
10930766	9.51	7.07	-2.44	0.18	25011	Cyp2c12	NM_031572
10932037	4.24	3.25	-0.99	0.50	313434	RGD1566 363	ENSRNOT00 000039809
10932139	5.06	4.04	-1.02	0.49	317170	Slc9a7	NM_0011082 42
10933772	3.74	2.81	-0.93	0.52	192267	Obp1f	NM_138903
10938524	6.71	5.08	-1.63	0.32	312102	Vsig4	NM_0010250 04

Table S3: List of common genes affected in brain and liver

(A) Common genes "down-regulated" in the brain and liver:

Gene Symbol	Accession No.	GO term molecular/biological functions
Wee1	NM_001012742	Protein tyrosine kinase activity
Slc2a12	NM_001107451	Carbohydrate transmembrane transporter activity
Hrk	NM_057130	Apoptosis regulation, Bleb assembly
Fam110b	NM_001024341.1	Tumor progression

(B) **Common genes "up-regulated" in the brain and "down-regulated" in the liver:**

Gene Symbol	Accession	GO term molecular/biological functions
Hba-a1	NM_013096	Heme binding, Oxidoreductase activity, Drug transport
Hba-a1	NM_013096	Heme binding, Oxidoreductase activity, Drug transport
Mup5	NM_203325.1	Fatty acid biosynthetic process, Catalytic activity

(C) **Common genes "up-regulated" in the liver and "down-regulated" in the brain:**

Gene Symbol	Accession	GO term molecular/biological functions
RT1-Da	NM_001008847	Response to stimulus
Zbtb6	NM_001108953	Transcriptional regulation
Tmem106b	NM_001004267	Dendrite morphogenesis, Required for dendrite branching

Table S4: Log fold change of gene expression after REM sleep loss compared to control*

Genes	Brain	Liver	Accession number
Slc1a4		1.46	NM_198763
Slc2a9		1.05	ENSRNOT00000042200
Slc2a12	-0.94	-1.08	NM_001107451
Slc5a7	-1.01		NM_053521
Slc6a6		1.17	NM_017206
Slc6a13		0.91	NM_133623
Slc9a4	-1.56		NM_173098
Slc9a2	-0.96		NM_012653
Slc15a3		0.88	NM_139341
Slc16a1		0.98	NM_012716
Slc16a14	-1.21		NM_001108229
Slc17a5		1.20	NM_001009713
Slc20a1		0.88	NM_031148
Slc22a5		1.37	NM_019269
Slc22a15	-0.89		NM_001107707
Slc25a22		0.98	NM_001014027
Slc27a2	1.07		NM_031736

Slc27a5		1.24	NM_024143
Slc28a2		2.02	NM_031664
Slc35d3		-1.05	NM_001107522
Slc39a5		1.03	NM_001108728
Slc39a12	-1.10		NM_001106124
Slc44a4		1.03	NM_212541

*(Values denoted with positive numbers shows upregulation while negative values show downregulation)

Table S5: List of GO terms related to synaptic plasticity

GO terms	Brain	RGD ID
Positive regulation of synaptic plasticity	DR (*p*=0.03)	GO:0031915
Positive regulation of long-term synaptic plasticity	UR (*p*=0.002)	GO:0048170
Regulation of long-term neuronal synaptic plasticity	DR (*p*=2.87E-005)	GO:0048169
Synaptic transmission	DR (*p*= 0.001)	
Synaptic transmission, dopaminergic	DR (*p*= 2.64E-009)	GO:0001963
Regulation of synaptic transmission, dopaminergic	DR (*p*= 0.0009)	GO:0032225
Negative regulation of synaptic transmission	DR (*p*= 0.004)	GO:0050805
Negative regulation of synaptic transmission, glutamatergic	DR (*p*=7.77E-009)	GO:0051967
Positive regulation of synaptic transmission, glutamatergic	DR (*p*=9.93E-006)	GO:0051968
Synaptic transmission, cholinergic	DR (*p*= 1.33E-006)	GO:0007271
Regulation of synaptic transmission, GABAergic	DR (*p*=2.47E-006)	GO:0032228
Positive regulation of synaptic transmission, GABAergic	DR (*p*=0.0002)	GO:0032230
Positive regulation of synaptic transmission, glutamatergic	DR (*p*=9.93E-006)	GO:0051968
Negative regulation of synaptic transmission, glutamatergic	DR (*p*=7.77E-009)	GO:0051967

Long term synaptic depression	DR (p=0.006)	GO:0060292
Long-term synaptic potentiation	DR (p=0.009)	GO:0060291
Neuromuscular synaptic transmission	DR (p=0.03)	GO:0007274
Negative regulation of synaptic transmission, dopaminergic	DR (p= 0.04)	GO:0032227
Establishment of synaptic specificity at neuromuscular junction	DR (p=0.04)	GO:0007529

DR=downregulated

Table S6: List of GO terms related to immune system

GO terms	Brain	Liver	RGD ID
Immune response		UR (p=0.0002)	GO:0006955
Immunoglobulin mediated immune response		UR (p=0.009)	GO:0016064
Negative regulation of type 2 immune response		DR (p=0.001)	GO:0002829
Regulation of immune system process		DR (p=0.01)	GO:0002682
Type 2 immune response		DR (p=0.01)	GO:0042092
Positive regulation of T cell mediated immune response to tumor cell	UR (p=0.0006)		GO:0002842
Immune system development	DR (p=0.001)		GO:0002520
T cell activation involved in immune response	DR (p=0.002)		GO:0002286

UR=upregulated, DR= downregulated
