# Supplementary figures and images for: Differential Gene Expression in Brain and Liver Tissue of Wistar Rats after Rapid Eye Movement Sleep Deprivation
Source: Clocks Sleep. 2020 Oct 23;2(4):442–65. doi: 10.3390/clockssleep2040033 (PMC7711450; doi:10.3390/clockssleep2040033)

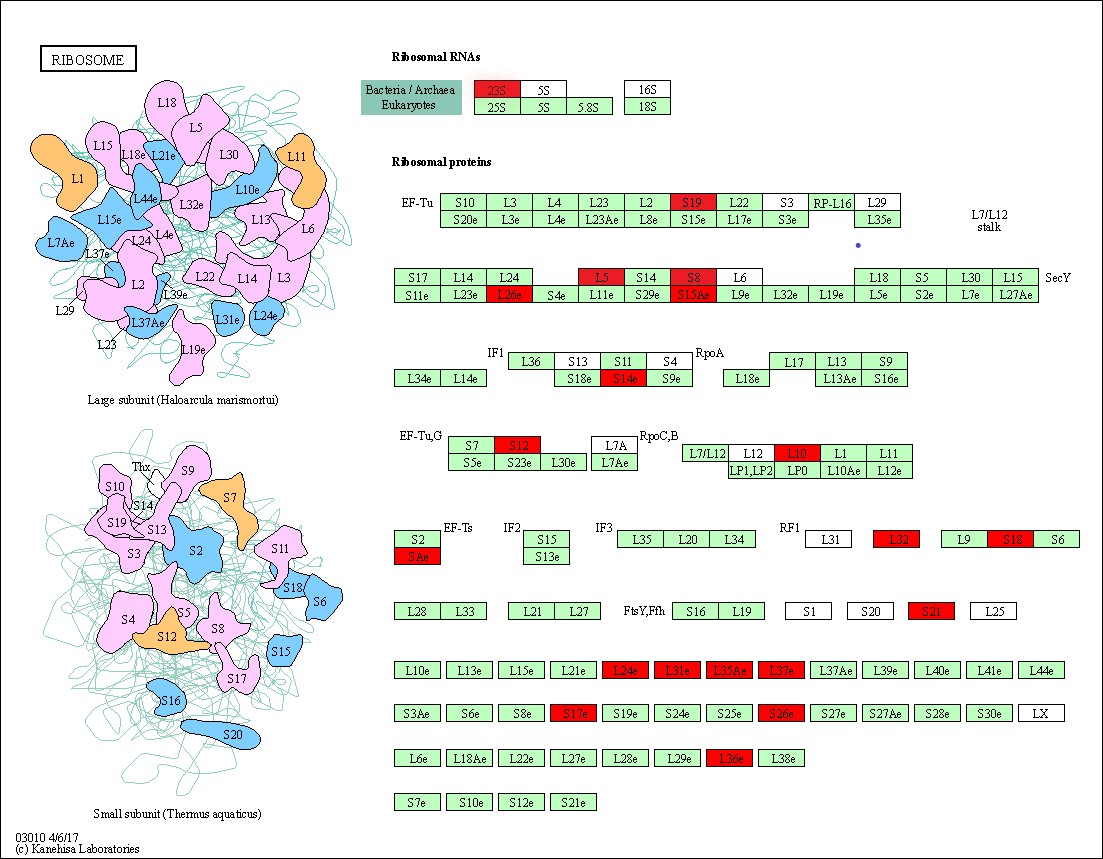

Supplement: Supplementary file 1 [file clockssleep-02-00033-s001.zip › Figure S1.jpg]

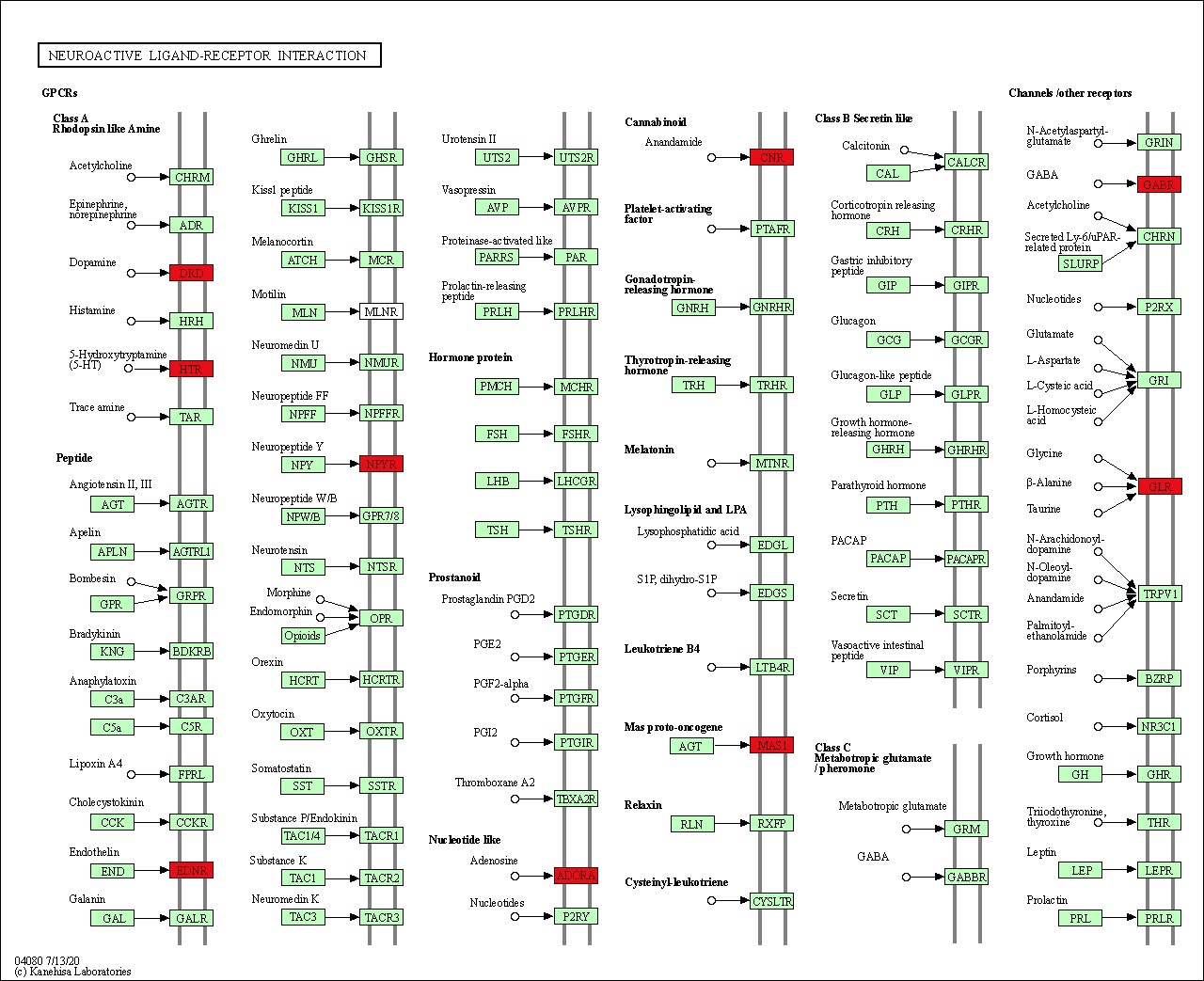

Supplement: Supplementary file 1 [file clockssleep-02-00033-s001.zip › Figure S2 .jpg]

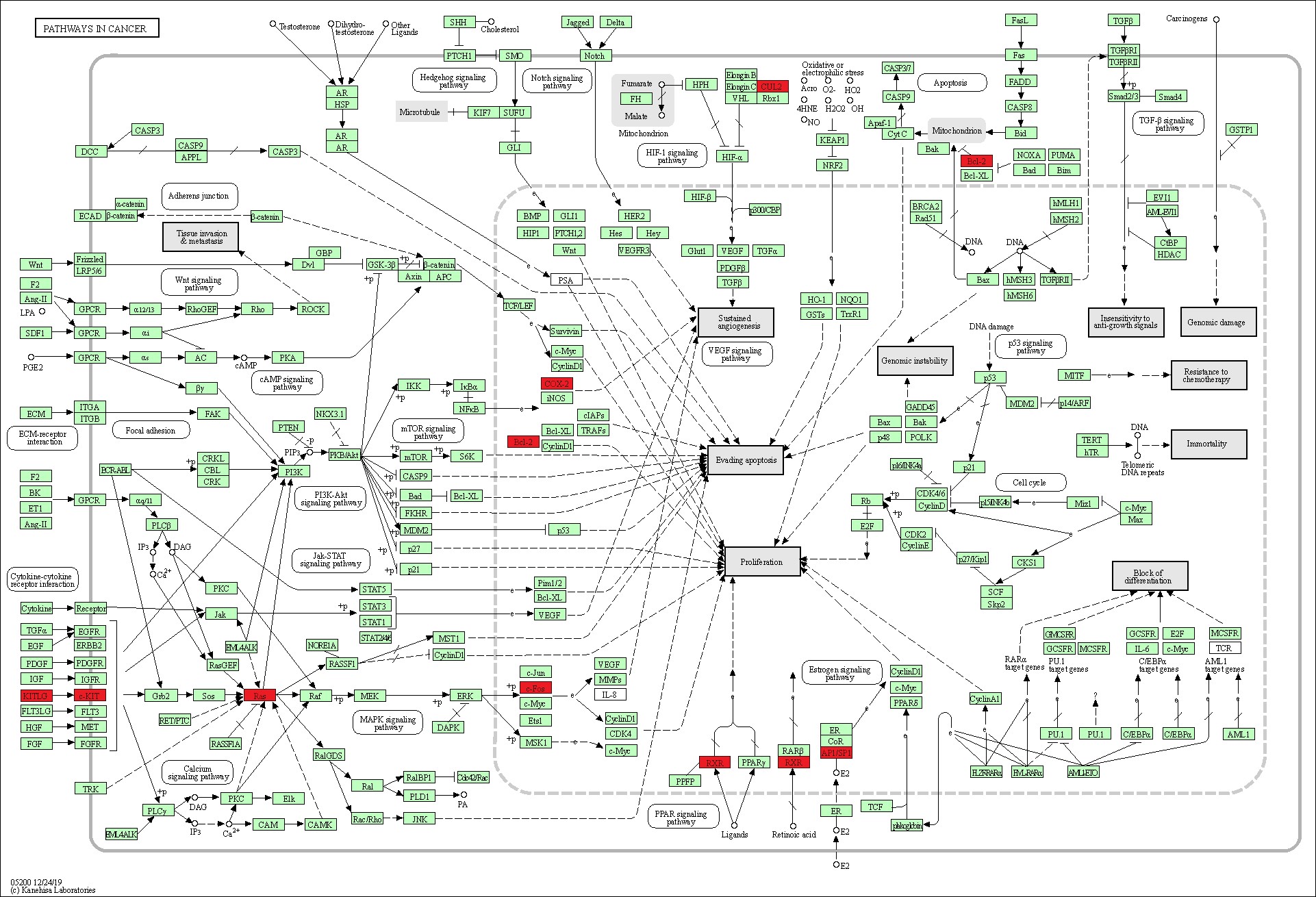

Supplement: Supplementary file 1 [file clockssleep-02-00033-s001.zip › Figure S3.jpg]

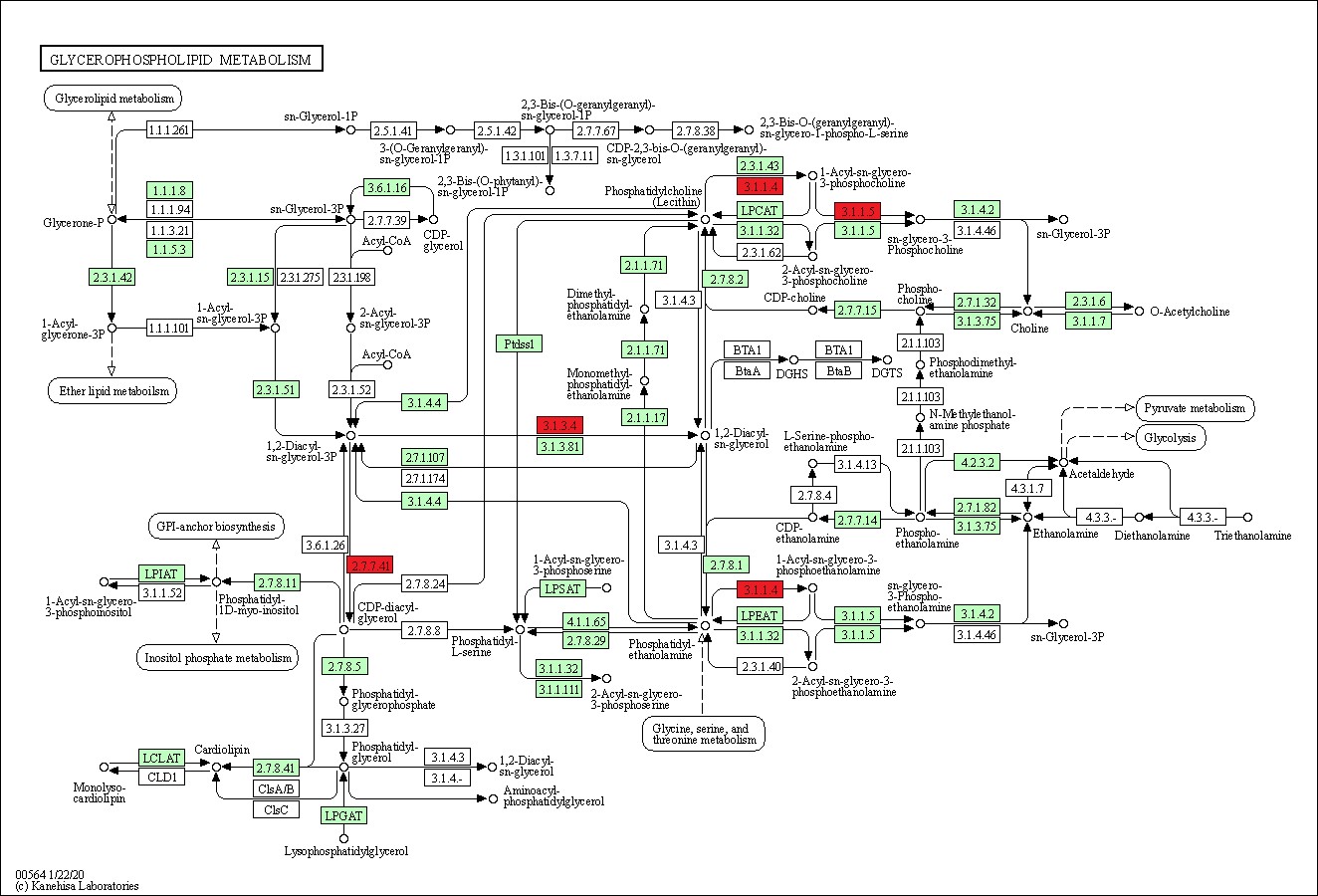

Supplement: Supplementary file 1 [file clockssleep-02-00033-s001.zip › Figure S4.jpg]
